# Realizing Multi-Parameter Measurement Using PT-Symmetric *LC* Sensors

**DOI:** 10.3390/s24206570

**Published:** 2024-10-12

**Authors:** Bin-Bin Zhou, Dan Chen, Chi Zhang, Lei Dong

**Affiliations:** 1 Hebei Key Laboratory of Electromagnetic Environmental Effects and Information Processing, Shijiazhuang Tiedao University, Shijiazhuang 050043, China; chendan@stdu.edu.cn (D.C.); zhangchi2015@stdu.edu.cn (C.Z.); 2 Key Laboratory of MEMS of the Ministry of Education, Southeast University, Nanjing 210096, China; dl@seu.edu.cn

**Keywords:** PT symmetry, *LC* sensor, multi-parameter measurement

## Abstract

With the rapid development in sensor network technology, the complexity and diversity of application scenarios have put forward more and more new requirements for inductor–capacitor (*LC*) sensors, for instance, multi-parameter simultaneous monitoring. Here, the parity–time (PT) symmetry concept in quantum mechanics is applied to *LC* passive wireless sensing. Two or even three parameters can be monitored simultaneously by observing the frequency response of the reflection coefficient at the end of the readout circuit. In particular, for three-parameter detection, a novel detection method is studied to extract the three resonant frequencies of the system through the phase–frequency characteristics of the reflection coefficient, which has never appeared in the previous literature on PT symmetry. The changes in three resonant frequencies are in response to changes in the three parameters in the environment. We show theoretically and demonstrate experimentally that the PT-symmetric *LC* sensor can realize multi-parameter measurement using a series LCR circuit as the sensor and a symmetric adjustable LCR circuit as the readout circuit. Our work paves the way for applying PT symmetry in multi-parameter detection.

## 1. Introduction

A passive wireless *LC* sensor is usually composed of a spiral inductor and a sensing capacitor, which are connected to form an *LC* resonator. The capacitor changes in response to the parameter of interest, resulting in the resonant frequency shift in the *LC* resonator. To wirelessly interrogate the *LC* sensor, an external readout coil is magnetically coupled with the sensor, and the resonant frequency of the sensor is detected through monitoring the input impedance or return loss of the readout coil. The intrinsic passive wireless properties make the *LC* sensor highly useful in situations where wired connections are difficult or even impossible [1], such as rotating parts and sealed environments. With the rapid development in the Internet of Things technology and the increasing application of portable and wearable devices [2,3], there is a demand to monitor multiple parameters simultaneously via a single chip, which requires miniaturization while achieving multi-functionalization.

In order to realize the effective integration of *LC* sensors with multiple parameters, researchers have explored a variety of schemes according to the design of *LC* sensor nodes and external readout circuits. Through utilizing the resonant frequency and the amplitude of the impedance, the measurement of temperature and humidity was performed through a single *LC* resonator [4]. However, this approach was limited to double-parameter detection and required known coupling coefficients. By stacking two individual *LC* resonators with each other, they could be used to monitor double parameters [5,6,7], leading to a limited measurement range due to the mutual coupling between the overlapping inductors. Then, a symmetrical dual-resonant circuit was proposed to optimize the above problem [8]; however, it greatly increased the complexity of the sensor structure. Multiple sensitive capacitors could be connected in parallel with a planar inductor, and the sensor could be controlled to operate in different modes using relay switches [9]. In theory, this scheme could achieve the simultaneous measurement of multiple parameters; however, the introduction of multiple active devices limited the application.

Different from conventional methods, this paper proposes theoretically and demonstrates experimentally that multi-parameter detection can be realized using a PT-symmetric *LC* sensing technique. PT symmetry, proposed in the context of quantum mechanics, means that a system is invariant under a combination of parity and time reversal operations [10]. The introduction of PT symmetry to classical wave dynamics can provide new design strategies for electronic devices [11]. A pair of inductively coupled *LC* resonators, one with amplification (gain) and the other with an equivalent amount of attenuation (loss), constitute a basic PT-symmetric system [12]. When applied to the *LC* wireless sensors, it can be used to enhance the sensing by operating under a PT-symmetric phase [13,14,15], near an exceptional point (EP) [16,17,18], or under a broken PT-symmetric phase [19]. However, to date, research has not found that the multi-resonant frequency characteristic of PT-symmetric systems in the exact phase can be used to achieve dual- or multiple-parameter sensing. In particular, this method maintains the characteristic of the sensor node as a simple single-resonant circuit.

## 2. Principles of PT-Symmetric Multi-Parameter Measurement

### 2.1. Dual-Parameter Measurement

**Figure 1:**
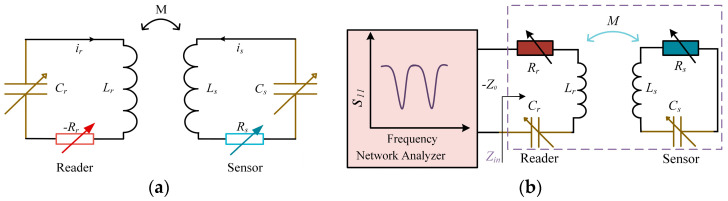

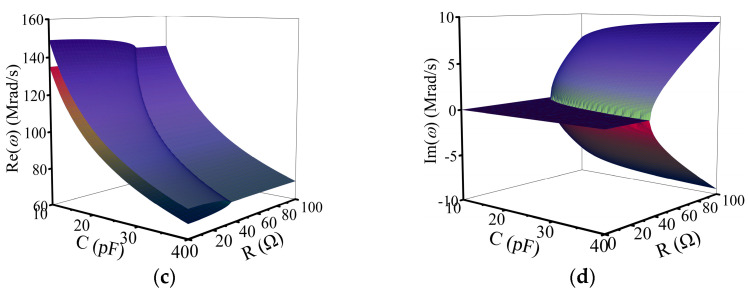
The PT-symmetric *LC* passive wireless multi-parameter sensing system. (**a**) The simplified circuit model of the PT-symmetric *LC* sensing system. (**b**) The equivalent circuit diagram of the PT-symmetrical *LC* sensing system for single-port measurement. (**c**) The real parts and (**d**) imaginary parts of the eigenfrequencies $\omega_{1,2}$ as a function of the varying capacitance and resistance, assuming *L* = 5 μH and *k* = 0.1.

Figure 1a presents a schematic diagram of the basic structure of the PT-symmetric *LC* passive wireless sensing system proposed in this work, showing a pair of inductively coupled LCR resonators, one with a gain (−*R*) and the other with an equivalent amount of loss (*R*). Since the *LC* sensor is a passive device with a positive resistance *R*s (usually a parasitic resistance of the inductor), it serves as the energy loss end. The other circuit has a negative resistor −*R*_r_, which provides energy as a readout circuit. An inductor *L*_s_ in series with a sensitive variable capacitor *C*_s_ and a sensitive variable resistor *R*_s_ can be a dual-parameter-sensitive sensor. To satisfy PT symmetry, it requires the relationship between the inductance, capacitance, and resistance in the two circuits: *L_r_* = *L*_s_ = *L*, *C_r_* = *C_s_* = *C,* and *R_r_* = *R_s_* = *R*. *M* is the mutual inductance between two inductors, $M=\frac{k}{\sqrt{LrLs}}=kL$, where *k* is the inductance coupling coefficient with a value between 0 (no coupling) and 1 (maximum coupling). In addition to the variable capacitor *C_s_* and the variable resistor *R_s_*, the coupling coefficient *k* can be used as the third sensitive variable parameter in the system.

Applying Kirchhoff’s law in the two series circuits,

(1) $$i\omega \left(LI_{r}+MI_{s}\right)-I_{r}R+\frac{I_{r}}{i\omega C}=0,$$

(2) $$i\omega \left(LI_{s}+MI_{r}\right)+I_{s}R+\frac{I_{s}}{i\omega C}=0,$$

where $I_{r}$ and $I_{s}$ are the currents in the gain (reader) and loss (sensor) tank, respectively, and ω is the system frequency. The natural frequency of the *LC* resonator is $\omega_{0}=1/\sqrt{LC}$. Equations (1) and (2) is rewritten as

(3) ω02−ω2−iωRL−kω2kω2ω02−ω2+iωRLIrIs=0.

From the above equation, this system satisfies PT symmetry, which means that Equation (2) remains unchanged when swapping the subscripts *r* and *s* (representing P inversion) and changing the polarity of the imaginary unit *i* (representing T inversion). This linear system has four eigenfrequencies,

(4) $$\omega_{1,2,3,4}=\pm \sqrt{\frac{2L-CR^{2}\mp \sqrt{{C^{2}R}^{4}-4LCR^{2}+4L^{2}k^{2}}}{2\left(1-k^{2}\right)L^{2}C}}.$$

We define the dimensionless non-Hermitian parameter or gain/loss parameter γ in the above system, $\gamma =R\sqrt{C/L}$.

(5) $$\omega_{1,2}=\frac{\sqrt{2-\gamma^{2}\mp \sqrt{\gamma^{4}-4\gamma^{2}+4k^{2}}}}{\sqrt{2\left(1-k^{2}\right)}}\omega_{0}$$

When $\gamma <\gamma_{EP}=\sqrt{2}\sqrt{1-\sqrt{\left(1-k^{2}\right)}}$, the system has two unequal real eigenfrequencies $\omega_{1,2}$ in the PT-symmetric exact phase.

When $\gamma =\gamma_{EP}=\sqrt{2}\sqrt{1-\sqrt{\left(1-k^{2}\right)}}$, the system eigenfrequencies $\omega_{1,2}$ coalesce at the exceptional point (EP).

When $\gamma >\gamma_{EP}=\sqrt{2}\sqrt{1-\sqrt{\left(1-k^{2}\right)}}$, the system eigenfrequencies $\omega_{1,2}$ are a pair of conjugate complex numbers in the PT-symmetric broken phase.

In order to obtain a clearer understanding of the variation in the eigenfrequencies with the capacitance and resistance, Figure 1c,d show the changes in the real and imaginary parts of the eigenvalues $\omega_{1,2}$, respectively, assuming *L* = 5 μH and *k* = 0.1. When there are two unequal frequencies in the real parts, the imaginary parts of the frequencies are 0, indicating that it satisfies $\gamma <\gamma_{EP}$ within this range of variation. In addition, as the resistance and capacitance vary, the low frequency $\omega_{1}$ and the high frequency $\omega_{2}$ both change significantly. When the resistance *R* increases, and the system satisfies $\gamma >\gamma_{EP}$, the dual real frequencies disappear in Figure 1c, and the imaginary parts of the frequencies begin to appear in Figure 1d. Therefore, the changes in the two unequal frequencies $\omega_{1,2}$ in the PT-symmetric exact region can be measured to reflect the changes in the sensitive resistance and capacitance parameters, ultimately realizing the purpose of passive wireless detection.

By decoupling Equation (5), the capacitance and resistance values can be obtained:

(6) $$C=\frac{1}{L\sqrt{1-k^{2}}\omega_{1}\omega_{2}},$$

(7) $$R=L\sqrt{{(\omega }_{1}^{2}+{\omega_{2}^{2})(k}^{2}-1)+2\omega_{1}\omega_{2}\sqrt{1-k^{2}}}.$$

Figure 1b shows the equivalent circuit model of a PT-symmetric system for single-port measurement. By connecting a vector network analyzer (VNA) to the readout circuit, the reflection coefficient *S*_11_ of the sensing system can be monitored, and the frequencies $\omega_{1,2}$ to be measured can be obtained. In the closed-loop analysis of the readout system, the VNA with a characteristic impedance *Z*_0_ of 50 Ω can be modeled as a negative resistance −*Z*_0_, as it provides energy to the system [13]. At the same time, in order to meet the PT symmetry of the system, the readout circuit adds a varying resistance *R*_r_ = *Z*_0_ − *R*_s_, so that the series equivalent total resistance is −*R*_s_ to match the varying resistance *R*_s_ = *R* in the sensor circuit. In addition, *L_r_* = *L_s_* = *L*, and *C_r_* = *C_s_* = *C*. The input impedance *Z_in_* at the port of the readout circuit is

(8) $$Z_{in}=j\omega L+\frac{1}{j\omega C}+\left(Z_{0}-Rs\right)+\frac{k^{2}L^{2}\omega^{2}}{j\omega L+\frac{1}{j\omega C}+R}.$$

The reflection coefficient *S*_11_, as a function of the input impedance *Z_in_*, is written as

(9) $$S_{11}={\left.\frac{Z_{in}-Z_{0}}{Z_{in}+Z_{0}}\right|}_{Z_{0}=50\Omega }=\frac{-R+\frac{k^{2}L^{2}\omega^{2}R}{{\left(\omega L-\frac{1}{\omega C}\right)}^{2}+R^{2}}+j(\omega L-\frac{1}{\omega C}+\frac{Ck^{2}L^{2}\omega^{3}(1-LC\omega^{2})}{1-2LC\omega^{2}+C^{2}\omega^{2}(R^{2}+L^{2}\omega^{2})})}{100-R+\frac{k^{2}L^{2}\omega^{2}R}{{\left(\omega L-\frac{1}{\omega C}\right)}^{2}+R^{2}}+j(\omega L-\frac{1}{\omega C}+\frac{Ck^{2}L^{2}\omega^{3}(1-LC\omega^{2})}{1-2LC\omega^{2}+C^{2}\omega^{2}(R^{2}+L^{2}\omega^{2})})}$$

Its amplitude in dB varies with the frequency:

(10) $$\left|S_{11}\right|=20\log\left|\frac{Z_{in}-Z_{0}}{Z_{in}+Z_{0}}\right|dB.$$

Differentiating the amplitude of *S*_11_ with the frequency *ω* in Equation (10), which is *∂|S*_11_*|/∂ω =* 0, the resonant frequencies $\omega_{l,h}$ at the minimum amplitude of *S*_11_ are obtained:

(11) $$\omega_{l,h}=\sqrt{\frac{2L-CR^{2}\mp \sqrt{{C^{2}R}^{4}-4LCR^{2}+4L^{2}k^{2}}}{2\left(1-k^{2}\right)L^{2}C}}.$$

It can be seen that the frequencies $\omega_{l,h}$ corresponding to the minimum value of *S*_11_ in Equation (11) are equal to the eigenfrequencies $\omega_{1,2}$ in Equation (4); so, the eigenfrequencies $\omega_{1,2}$ can be obtained through the amplitude–frequency curve of *S*_11_.

### 2.2. Three-Parameter Measurement

For the three-parameter measurement, the above two resonant frequencies cannot respond to the three unknown parameters. Therefore, this paper also proposes a new signal detection method for a PT-symmetric *LC* sensing system. By analyzing the phase–frequency characteristic of the single-port reflection coefficient *S*_11_, shown in Figure 1b, there are three resonant frequencies corresponding to the zero phase, which are related to the system parameters. Therefore, the three unknown parameters can be obtained by decoupling the three frequency equations, such as the capacitance, resistance, and coupling coefficient in the system. For practical applications in sealed spaces, in addition to the sensitive variable resistance and capacitance, the coupling coefficient corresponding to the coupling distance is usually unknown. At present, in the existing research, the coupling coefficient must be fixed and a known quantity; so, the detection method proposed in this paper can be used, which expands the application range of *LC* passive wireless sensors. The circuit model theory is the same as above, and we directly analyze the *S*-parameter phase–frequency characteristic.

Equation (9) has given the reflection coefficient *S*_11_ as a function of the frequency; so, its phase *ϕ* varies with the frequency as follows:

(12) $$\phi_{s_{11}}(\omega )=\tan^{-1}\frac{Im\left(s_{11}\right)}{Re\left(s_{11}\right)}.$$

By solving the equation *ϕ* = 0, the resonant frequencies corresponding to the zero phase of *S*_11_ are obtained:

(13) $$\omega_{0}=\frac{1}{\sqrt{LC}},$$

(14) $$\omega_{1,2}=\sqrt{\frac{2L-CR^{2}\mp \sqrt{{C^{2}R}^{4}-4LCR^{2}+4L^{2}k^{2}}}{2\left(1-k^{2}\right)L^{2}C}}.$$

It can be seen that the frequencies $\omega_{1,2}$ at the zero phase of *S*_11_ in Equation (14) are the system resonance frequencies $\omega_{1,2}$ in Equation (4), and an additional frequency $\omega_{0}$ in Equation (13) is the natural frequency of the *LC* resonator. By decoupling Equations (13) and (14), the system capacitance *C* and resistance *R*, as well as the coupling coefficient *k*, can be obtained:

(15) $$C=\frac{1}{L\omega_{0}^{2}},$$

(16) $$R=\frac{L\omega_{0}}{\omega_{1}\omega_{2}}\sqrt{2\omega_{1}^{2}\omega_{2}^{2}{-\omega }_{0}^{2}\omega_{1}^{2}{-\omega }_{0}^{2}\omega_{2}^{2}},$$

(17) $$k=\frac{\sqrt{\omega_{1}^{2}\omega_{2}^{2}-\omega_{0}^{4}}}{\omega_{1}\omega_{2}}.$$

## 3. Simulation

To verify that resonant frequencies of the system can be used to measure multiple sensitive parameters, the PT-symmetric *LC* sensing system was simulated using Agilent Software ADS (2020) with the circuit connection shown in Figure 1b. The sensor inductance coil *L_s_ =* 5 μH was connected to a variable capacitance *C*_s_ and variable resistance *R*_s_ to simulate the change in the sensitive capacitance and resistance in the experiment. To maintain PT symmetry, the inductance *L_r_* was set at 5 μH, and the variable capacitance *C_r_* and variable resistance *R_r_* in the readout circuit varied with the sensor. In addition, the coupling coefficient *k* was fixed at 0.1, and the sweep range was from 0 MHz to 30 MHz.

### 3.1. Dual-Resonant Frequencies

**Figure 2:**
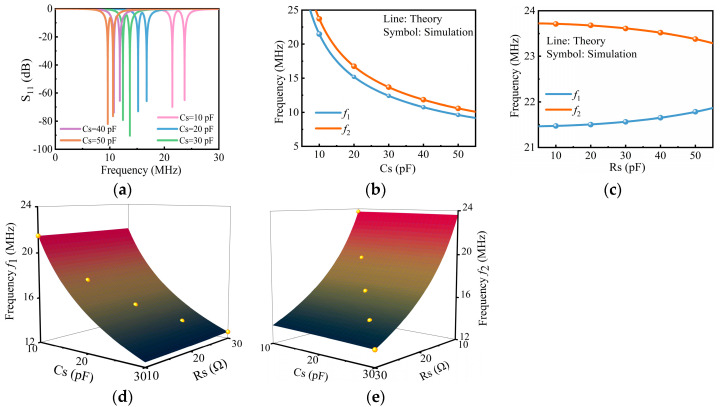
The frequency responses as a function of the sensitive capacitance and resistance. (**a**) The simulated reflection spectrum under different capacitances, with resistance *R*_s_ = 10 Ω. (**b**) The theoretical (blue and orange lines) and simulated (blue and orange symbols) frequency responses as a function of varying the capacitance *C*_s_, with the simulated results extracted from (**a**). (**c**) The theoretical (blue and orange lines) and simulated (blue and orange symbols) frequency responses as a function of varying the resistance *R*_s_, with *C*_s_ = 10 pF. The theoretical (surfaces) and simulated (yellow symbols) (**d**) frequencies *f*_1_ and (**e**) frequencies *f*_2_ responses as a function of varying the capacitance *C*_s_ and resistance *R*_s_.

First, keeping the resistance *R*_s_ = 10 Ω, the sensor capacitance *C*_s_ was changed from 10 pF to 50 pF with an interval of 10 pF, and the reader capacitance *C*_r_ kept changing synchronously. The changes in the two resonant peaks in the reflection coefficient *S*_11_ curve are shown in Figure 2a. Different color curves represent the *S*_11_ amplitude frequency curves under different capacitance values, with *f*_1_ and *f*_2_ corresponding to the low and high frequencies at the minimum values of the curves, respectively. Extracting the resonant frequencies from the simulated reflection spectrums in Figure 2a,b shows the resonant frequencies $f_{1,2}$ as a function of the varying capacitance values. As the capacitance value increased, both frequencies decreased simultaneously, and the simulated results (symbols) were consistent with the theoretical results (lines) shown in Figure 2b.

Then, keeping the capacitance *C*_s_ = *C*_r_ = 10 pF, the resistance *R*_s_ changed from 10 Ω to 50 Ω with an interval of 10 Ω, and the resistance in the readout circuit changed accordingly to retain the PT symmetry. The frequency responses as a function of varying the resistance *R*_s_ are shown in Figure 2c. The blue and orange symbols represent *f*_1_ and *f*_2_ under different resistance values, respectively, with *f*_1_ and *f*_2_ corresponding to the low and high resonant frequencies at the minimum of the simulated curves, respectively. From Figure 2c, it can be observed that as the resistance increased, the low frequency *f*_1_ increased, while the high frequency *f*_2_ decreased. The two peaks gradually approached each other, indicating a decrease in the splitting of *f*_1_ and *f*_2_. This phenomenon is due to the fact that the gain/loss parameter *γ* increased with the increase in the resistance. When *γ* increased to $\gamma_{EP}$, the eigenfrequencies *f*_1,2_ coalesced. The simulated results (symbols) were consistent with the theoretical analysis (lines).

Figure 2d shows the movement phenomenon of the dual frequencies with simultaneous changes in the capacitance and resistance. During the entire change process with the resistance and capacitance values varying from 10 to 30 with an interval of 5, the low frequency *f*_1_ changed from 21.47 MHz to 12.59 MHz, while the high frequency *f*_2_ changed from 23.71 MHz to 13.48 MHz. The simulated results (symbols) were consistent with the theoretical analysis (surfaces). The simulation results, shown in Figure 2d, verified that the dual-resonant frequencies *f*_1,2_ of the system can be used to measure the dual-sensitive parameters *R*_s_ and *C*_s_. By substituting the known inductance value *L* = 5 H, the coupling coefficient *k* = 0.1, and the simulated results *f*_1_ = *ω*_1_/2π = 21.471 MHz and *f*_2_ = *ω*_2_/2π = 23.713 MHz into Equations (6) and (7), the calculated capacitance value *C*_s_ = 10.000 pF and the resistance value *R*_s_ = 10.050 Ω with a maximum relative error of 0.5% were obtained. The error mainly comes from the accuracy of the extracted resonant frequencies *f*_1,2_, which is related to the frequency scanning step of the instrument. The smaller the step, the smaller the error.

### 3.2. Three Resonant Frequencies

We simulated the PT-symmetric *LC* sensing system to verify that the three resonant frequencies $f_{0,1,2}$ can be obtained by the phase–frequency characteristic curve of the reflection coefficient *S*_11_ and used to measure the three sensitive parameters *R*_s_, *C*_s_, and *k*. Setting the inductance value to 5 μH, the capacitance varied from 10 pF to 50 pF and the resistance increased from 5 Ω to 45 Ω.

In order to ensure that the entire range of changes was within the PT-symmetric exact phase, it was necessary to meet the requirement $\gamma <\gamma_{EP}$; that is,

(18) $$R\sqrt{\frac{C}{L}}<\sqrt{2-2\sqrt{1-k^{2}}}.$$

Therefore, *k* should vary within a range, $k>\frac{9\sqrt{15919}}{8000}$. The range of *k* variation was set from 0.15 to 0.35, with an interval of 0.1. The coupling coefficient *k* was not set as very large to avoid significant frequency splitting between the two frequencies in $\omega_{1,2}$, in order to minimize the scanning frequency range and improve the measurement accuracy as much as possible.

**Figure 3:**
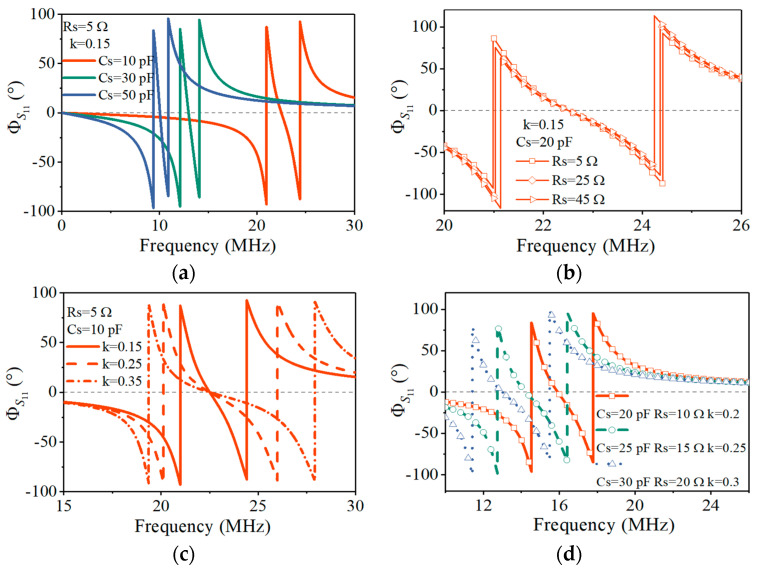
Simulated frequency responses as a function of varying the parameters. (**a**) Reflection spectrums under different capacitances. (**b**) Reflection spectrums under different resistances. (**c**) Reflection spectrums under different coupling coefficients. (**d**) Reflection spectrums as a function of varying the capacitance *C*_s_, resistance *R*_s_, and coupling coefficient *k*.

Firstly, keeping the resistance *R*_s_ = 5 Ω and the coupling coefficient *k* = 0.15 unchanged, the sensor capacitance *C*_s_ changed from 10 pF to 50 pF at 20 pF intervals, and the reader capacitance *C*_r_ followed its changes. The variation in the *S*_11_ phase with the frequency is shown in Figure 3a. Each color curve corresponds to a fixed capacitance. The zero-phase horizontal gray dashed line has three intersection points with each curve, indicating that there were three resonant frequencies corresponding to the zero phase. As the capacitance increased, the curve shifted to the left, and the three resonant frequencies $f_{0,1,2}$ gradually decreased at the same time. The change in capacitance *C*_s_ affected the three frequencies simultaneously, which was consistent with the theoretical analysis in Equation (13).

Then, keeping the capacitance fixed at *C*_s_ = 10 pF and the coupling coefficient *k* = 0.15 unchanged, the sensor resistance *R*_s_ changed from 5 Ω to 45 Ω at 20 Ω intervals, and the reader resistance *R*_r_ followed it to change with 50-*R*_s_ to satisfy the PT symmetry. The changes in the three resonant frequencies $f_{0,1,2}$ are shown in Figure 3b. The different symbol curves represent the simulated results under different resistances. As the resistance increased, the middle resonant frequency *f*_0_ remained fixed, while the other two resonant frequencies, *f*_1_ and *f*_2_, were close to each other, indicating that the resistance *R*_s_ did not affect *f*_0_ but only reduced the $f_{1,2}$ splitting. This result is also consistent with the theoretical analysis in Equation (13) and Figure 1c. When the resistance continued to increase, and inequality (16) became an equation, the frequencies *f*_1_ and *f*_2_ coalesced, indicating that the system was at EP.

Next, keeping the capacitance *C*_s_ = 10 pF and resistance *R*_s_ = 5 Ω unchanged, the coupling coefficient *k* changed from 0.15 to 0.35 at a 0.1 interval. Figure 3c shows the variation in the three resonant frequencies with the coupling coefficient *k*. Different style curves represent the simulated results under different coupling coefficients. As the coupling coefficient increased, the intermediate resonant frequency *f*_0_ remained fixed, while the other two resonant frequencies $f_{1,2}$ stayed away from each other, indicating that the coupling coefficient *k* did not affect *f*_0_ but only increased the $f_{1,2}$ splitting. The larger the *k* value, the more the two resonant frequencies *f*_1_ and *f*_2_ were separated.

Finally, Figure 3d shows the frequency responses as a function of the sensitive capacitance, resistance, and coupling coefficient. By substituting the known inductance value *L* = 5 μH and the simulated results *f*_1_ = *ω*_1_/2π = 14.54 MHz, *f*_0_ = *ω*_0_/2π = 15.92 MHz, and *f*_2_=*ω*_2_/2π = 17.79 MHz corresponding to the orange curve into Equations (15)–(17), the calculated capacitance value *C*_s_ = 19.989 pF, resistance value *R*_s_ = 9.384 Ω, and coupling coefficient *k* = 0.2 were obtained. Compared with the theoretical values *C*_s_ = 20 pF, *R*_s_ = 10 Ω, and *k* = 0.2, the resistance had the maximum relative error of 6.16%, which mainly came from the limited accuracy of the extracted resonant frequencies $f_{1,2}$. The measurement accuracy is related to the simulation scanning step. The smaller the step and the more scanning points within the same scanning frequency range, the smaller the error. The simulation results verified that the three resonant frequencies $f_{0,1,2}$ of the system can be used to measure the three sensitive parameters *R*_s_, *C*_s_, and *k*.

## 4. Experimental Results and Discussion

**Figure 4:**
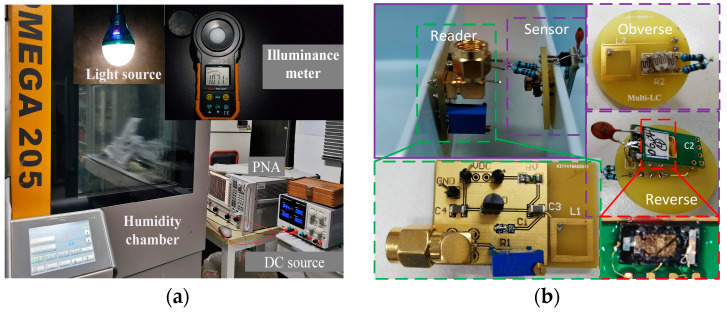
The experimental setup of the PT-symmetric *LC* wireless sensor. (**a**) Experimental instruments; (**b**) experimental circuits.

**Figure 5:**
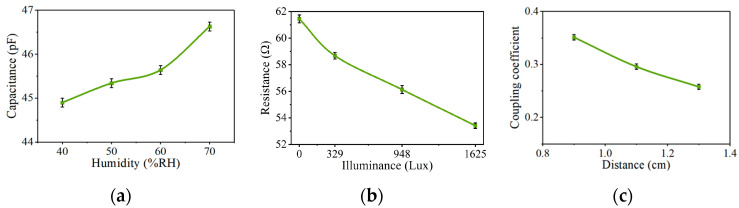
Measured variation in the sensitive parameters with the environment. (**a**) The variation in the capacitance *C*2 value with humidity. (**b**) The variation in the resistance *R*2 value with illuminance. (**c**) The variation in the coupling coefficient *k* with the coupling distance *d*.

In order to demonstrate our results, we built the prototype shown in Figure 4b using onboard circuit technology. The experimental setup is shown in Figure 4a, including a humidity chamber (OMEGA 205), a light source (LED bulb), an illuminance meter (PM6612), a vector network analyzer (Agilent N5224A PNA), a DC voltage source, and other components. In the experiment, a humidity-sensitive capacitor fabricated by MEMS technology and a photoresistor were used to detect the environmental humidity, light intensity, and inductive coupling distance (sensor position). Figure 4b shows the actual experimental circuits. The sensor inductor *L*2 was a PCB planar spiral inductor with a substrate thickness of 1.5 mm, containing six coils, an outer diameter of 10 mm, a line width of 150 μm, and a line spacing of 100 μm. The humidity-sensitive capacitor *C*2 was a preliminary achievement of our research group, which is a sandwich structure implemented using MEMS technology, and the moisture sensing medium was graphene oxide. The variation in the capacitance *C*2 value with the humidity is shown in Figure 5a. The photoresistor used a commercial product with the model number 5537. The resistance value of the photoresistor varied within the range of 20~50 kΩ with the light intensity. To reduce the resistance value, three photoresistors were connected in parallel with two low resistances as the sensitive resistor *R*_2_. The variation in the resistance *R*2 value with the light intensity is shown in Figure 5b. The inductor *L*2, sensitive capacitor *C*2, and sensitive resistor *R*2 were connected in series to form an LCR resonant circuit as an *LC* sensor. It is worth noting that due to the parallel connection of small resistors with sensitive resistors in the LCR circuit, large changes in the light intensity caused relatively small changes in the resistance, as shown in Figure 5b, resulting in small frequency changes. Therefore, in practical application scenarios, a sensitive resistor with a reasonable range in variation should be selected.

Correspondingly, the design of inductance *L*1 was the same as that of the sensor inductance *L*2, and a variable capacitance diode with model BB910 was connected as the variable capacitor in the readout circuit. The DC voltage source, connected in parallel at both ends of the varactor diode, controlled the capacitance value. *C*3 and *C*4 were decoupling capacitors with a capacitance of 1 μF, which were used to isolate the impact of the DC voltage on the circuit. The resistance RV equal to 1 MΩ was used to prevent the RF signals from the readout circuit entering the DC voltage source. The variable resistor *R*1, connected to the other end of the diode, could be adjusted to match the sensor resistor *R*2. We connected the readout circuit port to a vector network analyzer with an output power of 20 dBm, and scanned from 13 MHz to 17 MHz in 6 KHz steps. The sensor and readout circuit were fixed on two movable supports. In the experiment, the coupling coefficient of the system was changed by manually adjusting the coupling distance between the two inductors, whose relationship is shown in Figure 5c.

During the measurement, the sensor was placed in the humidity chamber, which could be manually operated to set the humidity of the environment inside the chamber. A wireless adjustable-brightness LED bulb was placed in a humidity chamber as a light source to change the light intensity in the environment. Meanwhile, an illuminance meter was used to calibrate the illuminance in the humidity chamber. The VNA was used for the frequency measurement of the *LC* sensors.

**Figure 6:**
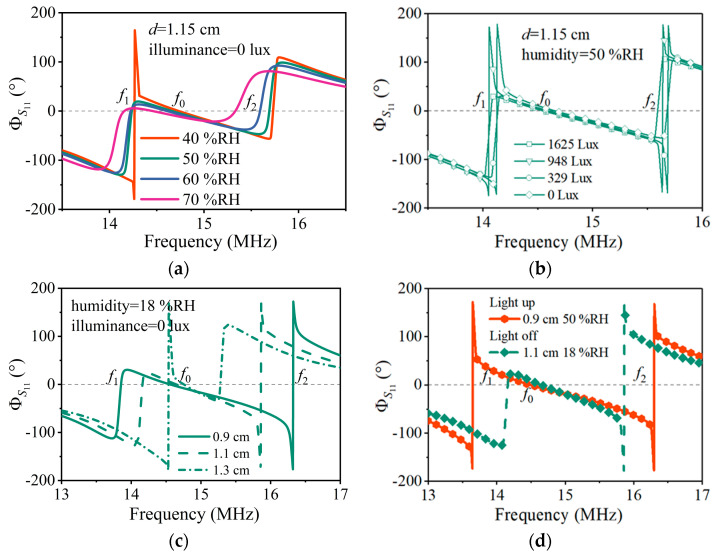
Measured frequency responses as a function of the sensitive parameters. (**a**) Reflection spectrums with different relative humidities. (**b**) Reflection spectrums with different illuminances. (**c**) Reflection spectrums with different coupling distance. (**d**) Reflection spectrums as a function of varying the relative humidity, illuminance, and coupling distance.

Firstly, the humidity was increased from 40% RH to 70% RH at intervals of 10% RH, while the illumination was set to 0 lux, and the coupling distance was maintained at 1.15 cm. The *S*_11_ phase–frequency characteristic curves obtained through VNA are shown in Figure 6a. In the figure, different colored curves represent the measurement results under different relative humidities. Each curve intersects with the zero-phase line (gray dashed line) at three points, and the resonant frequencies of the left, middle, and right intersection points correspond to $f_{1}$, $f_{0}$, and $f_{2}$ in the above analysis, respectively. When humidity changes caused capacitance changes, all three resonant frequencies changed, which was consistent with the trend in the simulated result, shown in Figure 3a.

Then, the lighting intensity of the LED bulb changed from 0 lux to 1625 lux, while the humidity was kept at 50% RH, and the coupling distance was maintained at 1.15 cm. The *S*_11_ phase frequency characteristic curves are shown in Figure 6b. Different symbol curves represent the measurement results under different illumination intensities. The resonant frequencies $f_{1}$ and $f_{2}$ changed with the lighting intensity, and $f_{0}$ remained constant, because the light intensity caused a change in the resistance *R*2 independent of $f_{0}$, which was consistent with the theoretical and simulated results, shown in Figure 1c and Figure 3b.

Next, the coupling distance between the two inductive coils was manually adjusted to change from 0.9 cm to 1.3 cm with a spacing of 0.2 cm, while the room humidity was kept at 18% RH, and the illumination was maintained at 0 lux. The experimental results provided the *S*_11_ phase–frequency characteristics, as shown in Figure 6c. Different style curves represent the measurement results under different coupling distances. As the distance changed, the resonant frequencies *f*_1_ and *f*_2_ had significant changes, while *f*_0_ in the middle remained basically unchanged, which was consistent with the simulation results shown in Figure 3c. The error was due to the parasitic effects.

Finally, the two curves shown in Figure 6d are the measurement results under different light intensities, relative humidities, and coupling distances. As the three parameters changed simultaneously, all three resonant frequencies shifted, which agreed qualitatively with the theoretical and simulated results shown in Figure 3d.

## 5. Conclusions

In conclusion, we theoretically analyzed and simulated the PT-symmetric circuit, and the results showed that the system has two real resonant frequencies related to the circuit parameters in the PT-symmetric exact phase. The frequencies corresponding to the two minimum values of the amplitude frequency characteristic curve of the *S*_11_ parameter measured by the single port of the readout circuit are the two resonant frequencies of the system. Two frequency equations can be decoupled to obtain two unknown circuit component parameter values. Therefore, it can be applied to the dual-parameter measurement of *LC* passive wireless sensors, and the measured changes in the dual-resonant frequencies of the system are used to respond to the changes in the sensitive component parameters of the two systems caused by the environment. On the basis of the above, it was further discovered that the zero phase of the measured *S*_11_ phase frequency characteristic curve corresponds to three resonant frequencies; therefore, three frequency equations can be solved to obtain three system parameters, including the capacitance, resistance, and coupling coefficient, realizing the simultaneous measurement of the three parameters. Finally, the sensitivity of the humidity, illuminance, and distance parameters was experimentally verified using a PT-symmetric *LC* passive wireless sensing system. The results presented here provide a feasible new approach for realizing PT-symmetric *LC* passive wireless multi-parameter sensing.

## Data Availability

Data is contained within the article.
